# Development and validation of an assessment tool for public health emergency management program

**DOI:** 10.1186/s41256-025-00423-z

**Published:** 2025-09-23

**Authors:** Sileshi Demelash Sasie, Getinet Ayano, Medhanit Girma, Pien Van Zuylen, Fantu Mamo Aragaw, Tadele Dana Darebo, Lorena Guerrero-Torres, Afework Mulugeta, Mark Spigt

**Affiliations:** 1 Ethiopian Public Health Institute, Addis Ababa, Ethiopia; 2 Department of Family Medicine, CAPHRI School for Public Health and Primary Care, Maastricht University, Maastricht, The Netherlands; 3 School of Population Health, Curtin University, Perth, Australia; 4 Medical and Health Science College Ethiopia, University of Gondar, Gondar, Ethiopia; 5 Alliance for Health Policy and Systems Research, Science Division, World Health Organization, Geneva, Switzerland; 6 Medical and Health Science College, Mekele University, Mekele, Ethiopia; 7 General Practice Research Unit, Department of Community Medicine, UiT the Arctic University of Norway, Tromsø, Norway

**Keywords:** Instrument psychometrics, PHEM, Programs, Tool development, Validation

## Abstract

**Background:**

Effective public health emergency management (PHEM) is crucial for safeguarding population health and societal resilience in the face of escalating global threats. However, standardized tools for comprehensively assessing emergency readiness across diverse contexts are lacking, particularly in resource-constrained settings like Ethiopia. Existing assessment approaches have demonstrated limitations, including narrow scope focused on specific hazards or sectors, over-emphasis on implementation processes rather than programmatic outcomes, and lack of empirical grounding in tool development and validation. This study aimed to develop and validate a standardized tool to assess PHEM programs.

**Methods:**

This study employed a sequential exploratory mixed methods design. Relevant assessment domains were identified through a literature review, stakeholder consultations, and expert consultations conducted at a workshop. This study utilizes Donabedian's structure-process-outcome framework to guide the assessment of public health emergency management**.** A questionnaire containing 60 items was then generated and underwent translation, face validation, and content validity assessment. Construct validity was evaluated using exploratory factor analysis with responses from 260 professionals. Internal consistency reliability was assessed utilizing Cronbach's alpha.

**Results:**

A 45-item tool for assessing PHEM programs in diverse contexts in Ethiopia was developed and validated. The tool demonstrated high content validity (CVIs > 0.83), good construct validity (15-factor structure explaining 74.8% variance), and excellent reliability (overall α = 0.863, subscales > 0.70). The final tool covers domains such as multi-sectoral coordination, resource allocation, transparency/accountability, workforce capacity, and provision of essential supplies.

**Conclusions:**

This study developed a comprehensive tool to assess emergency management programs across diverse contexts. The validation revealed critical preparedness factors like multi-sector coordination, funding transparency and workforce strength. The mixed-methods approach proved effective for crafting contextually appropriate evaluation methods in low-resource settings with infrastructure barriers. By standardizing measurement of capacities and gaps, this validated tool can guide strategic policy planning to bolster resilience nationwide. Ongoing monitoring of progress using this model can help prioritize investments and direct coordinated responses to future crises.

**Supplementary Information:**

The online version contains supplementary material available at 10.1186/s41256-025-00423-z.

## Background

Public health emergencies increasingly threaten low-income countries. From 1994 to 2015, weather-related disasters caused over 1.5 million deaths and affected over 4 billion people globally [1]. The COVID-19 pandemic led to over 777 million cases and 7 million deaths by early 2022, revealing health systems'vulnerabilities [2]. Past epidemics, like the 2013–2016 West Africa Ebola outbreak, resulted in over 28,000 cases and 11,000 deaths [3]. Low-income nations face up to 91% of weather-related disaster mortality and bear disproportionate burdens from climate change impacts [4].With growing risks from urbanization, conflict, and climate change, low-resource communities are increasingly vulnerable to public health emergencies [5–7].

Developing robust emergency management programs is challenging for low-income nations due to barriers like insufficient workforce, limited funding, weak governance, and poor infrastructure [8–10]. This issue is evident in Ethiopia, where malaria, emergent diseases like COVID-19, and disruptions from manmade and natural hazards threaten population health [10]. hile Ethiopia serves as the primary case study, the tools developed here have broader implications for improving emergency preparedness in other low-resource settings globally.

PHEM integrates knowledge, techniques, and principles for managing complex health events [11]. The field continues to evolve, drawing lessons from recent global emergencies to propose frameworks for strengthening health resilience [11]. Assessing the readiness of PHEM programs is essential for identifying barriers and guiding resource allocation [12]. However, past efforts have faced inconsistent tools and methodologies [12]. However, past efforts have faced inconsistent tools, methodologies [8], with some lacking a holistic, multidisciplinary perspective, often focusing on specific sectors or hazards [12–14]. Studies show a lack of rigor in assessing effectiveness and best practices [12]. While some tools collect systematic data, they may not capture programmatic impact [12, 13].

Local assessments have demonstrated the value of regular evaluations but also exposed shortfalls such as deficient workforces, infrastructure, resources, and intersectoral collaboration [15–17]. Assessments in South Asia and the Middle East revealed weaknesses in vector control programs, including poor surveillance and insecticide resistance monitoring [18]. A global study on disease surveillance identified fragmentation between sectors and resource constraints as common challenges [19]. The implementation of One Health approaches in antimicrobial resistance surveillance faces similar obstacles, requiring improved collaboration and equity [20]. hese findings highlight the need for comprehensive, context-specific evaluations of emergency preparedness to optimize governance and strengthen protection against public health emergencies globally [8].

Existing research on PHEM programs is often limited in scope, focusing on single hazards and neglecting an integrative perspective. Current assessments prioritize specific aspects over holistic outcomes, resulting in fragmented evaluations. The lack of standardized evaluation tools complicates systemic assessments of effectiveness, hindering meaningful conclusions. This study identifies gaps in resources (structure), processes (coordination), and outcomes within the PHEM framework based on Donabedian's model [21, 22].

To address these gaps, this study aims to develop and validate a standardized assessment tool that evaluates various aspects of program implementation, including readiness, response capabilities, and overall effectiveness in the context of epidemics (e.g., COVID-19, Ebola) and natural disasters (e.g., floods, droughts). By employing Donabedian's framework, which categorizes evaluations into structure, process, and outcomes [23], the study will enhance understanding of infrastructure, response activities, and public health impact [24]. The anticipated outcomes include informing policy decisions, improving resource allocation, and guiding strategic planning to strengthen public health emergency response capabilities in low-resource settings.

## Methods

### Study setting, period design

The study was conducted in Ethiopia, a country in the Horn of Africa with a population of about 130 million. It is the second most populous nation in Africa and is bordered by Eritrea to the north, Djibouti and Somalia to the east, Sudan and South Sudan to the west, and Kenya to the south. The study purposefully engaged diverse national, regional, and local emergency management practitioners across Ethiopia's heterogeneous contexts. The study period was from July 2023 to June 2024.

During this period, the literature and desk review informing the initial tool design was conducted over approximately six months, starting from July 2023. An initial draft questionnaire was developed within one month following this preliminary work. Stakeholder feedback was incorporated through consultation workshops over one month. Face and content validation was completed in one month, followed by nearly two months for pilot testing. In total, twelve months were dedicated to instrument development, expert input, and finalizing the tool following validity testing.

### Public health models: Donabedian framework

The literature review addressed the structural dimension of the Donabedian framework by identifying critical gaps in resources, infrastructure, and supply chains essential for PHEM, thereby informing the design of our assessment tool. Our methodology included consultative workshops with key stakeholders to uncover procedural challenges like ineffective communication and limited inter-sectoral coordination, which helped establish best practices and collaborative approaches for effective PHEM. The outcome dimension was validated through exploratory factor analysis face validity, and content validity assessments, ensuring the tool accurately captures key PHEM components, thereby measuring the effectiveness of emergency management programs. We also demonstrated how these theoretical components apply in practice, with elements like resource allocation and multi-sectoral coordination directly enhancing emergency response times and community resilience, ultimately improving public health outcomes.

### Instrument development procedures

Instrument development followed a comprehensive process to establish a robust theoretical and empirical foundation. A literature review synthesized relevant frameworks, defining core domains and indicators. Subject matter experts conducted a focused evidence review, evaluating conceptual coherence and extracting measurement priorities. An expert consultative workshop employed structured techniques optimizing content validity and applicability. The conceptual model iteratively evolved by integrating stakeholder viewpoints and triangulating evidence sources into a contextually grounded, multidimensional framework. Items were systematically developed based on psychometric principles to assess hypothesized theoretical constructs and associations across proposed subscales. Finally, rigorous translation protocols involving independent forward and back translation addressed linguistic equivalence prior to pilot testing.

Additionally, we have incorporated Donabedian's Structure-Process-Outcome Model as a theoretical framework to guide the development and validation of our tool. This model allows us to assess the resources and workforce (structure), the multi-sector coordination and resource allocation processes (process), and the effectiveness of responses and community resilience [25]. By employing this framework, we ensure that our tool not only assesses the implementation status of public health emergency management programs but also elucidates the relationships between structures, processes, and outcomes. This theoretical underpinning enhances the comprehensiveness of our tool and its effectiveness in both local and global contexts.

### Literature review and desk review

The study followed methods described by Zamanzadeh et al. [26] to develop the questionnaire. To establish a theoretical foundation, an extensive literature review was conducted by a team of eight subject matter experts [27]. This review systematically identified, evaluated, and synthesized relevant empirical studies and theoretical frameworks from the published literature on public health emergency management and program assessment, following best practices [28, 29]. Specifically, the literature review explored existing frameworks and models for public health emergency management programs; core components and domains of emergency preparedness and response systems; items and factors influencing program implementation status; validated assessment tools used to evaluate emergency management programs; and relevant population, implementation, and outcome-related variables. This review process defined pertinent domains and key items, and identified important variables, populations, and validated domains for capturing multi-level determinants of public health emergency management program implementation status to inform development of the assessment tool. While resources and processes are critical, the outcomes associated with effective PHEM programs remain underexplored. This study aims to bridge this gap by integrating outcome measures aligned with Donabedian's framework.

A focused desk review was conducted in Adama town by subject matter experts [30] to critically appraise and consolidate the evidence synthesized during the comprehensive literature review phase. This process involved evaluating the conceptual coherence, empirical grounding, and contextual relevance of identified domains and indicators [31], cross-analyzing themes to ensure theoretical saturation [32], and extracting key measurement priorities and substantive focal areas for tool development [33]. The desk review facilitated systematic consolidation of the accumulated knowledge and distillation of the evidence into a coherent preliminary framework. This framework synthesized diverse perspectives to advance the nascent measurement model by comprehensively integrating empirical findings and conceptual underpinnings. Through an iterative process, the output was refined to subsequently inform consultative workshop discussions. These discussions centered on examining the proposed items'conceptual relationships, the underlying constructs being assessed, and strategies for optimizing the tool's applicability across various contexts.

### Consultative workshop

A two-day consultative workshop was then held in Hawassa, the southern capital of Ethiopia, where we brought together 12 subject matter experts from all regions of the country. Participants included public health officials who provided local contextual insights, as well as national experts from organizations such as the Ethiopian Public Health Institute and the Ministry of Health, who contributed a broader perspective. The workshop employed various techniques to gather stakeholder input aimed at enhancing the content validity of the measurement tool. An iterative framework development phase systematically integrated these diverse Ethiopian viewpoints. Guided by this optimized theoretical model, we developed initial items for pilot testing, taking into account evidence from previous steps that engaged national and regional Ethiopian experts. Overall, this multi-step instrument development process embedded perspectives specifically from within the Ethiopian public health context, ensuring the cultural relevance and local applicability of the tool for use in Ethiopia.

Item review and refinement utilized a mixed deductive-inductive approach [34]; Q-sort procedures assessed convergent and discriminant validity [35]; focus groups examined item clarity, relevance, and comprehensiveness [36]; and expert review with content validity indexing was conducted [37]. Through iterative consensus-building, experts critically evaluated the conceptual relationships between proposed items and underlying constructs, probing construct validity, semantic appropriateness, and theoretical congruence of the initial item pool. Contextualization strategies were devised to enhance cultural relevance, community acceptability, and local applicability, recommending systematic translation procedures incorporating back-translation, cognitive debriefing, and pilot testing to ensure conceptual and semantic equivalence [38]. This structured consultative process leveraged complementary qualitative and quantitative approaches to critically examine the emerging instrument's psychometric properties, consolidating multidisciplinary inputs to optimize content validity, construct validity, and cross-cultural applicability prior to pilot testing and psychometric evaluation.

### Iterative framework development

The iterative framework development process systematically integrated diverse stakeholder viewpoints and empirical evidence from multiple sources to advance the nascent measurement model. It involved triangulation across findings from the literature review, desk review, and consultative workshop [39], critical reflection and theory-based refinement after each development stage [40], constant comparative analysis to identify convergent and divergent perspectives [41], and member checking to verify interpretive congruence with participant intents [42]. This iterative approach facilitated critical examination of the consolidated conceptual, empirical, and methodological evidence, with feedback informing revisions to optimize alignment between the evolving theoretical model and empirical data [43]. By synthesizing inputs from a multidisciplinary team of five core researchers engaging 10 external experts/stakeholders across phases like expert reviews, the comprehensive process enhanced the construct validity and content validity of the final integrated measurement framework [31].

### Item development

Item generation was guided by established scale development procedures and best practices from the psychometric literature. Measurement items were drafted to logically assess the identified theoretical factors and their hypothesized relationships [31, 34]. This involved grouping items into proposed subscales corresponding to the key construct domains [44]. Item formulation carefully considered evidence from the literature review, expert consultations with a panel of 10 subject matter experts [30], and stakeholder inputs to ensure content representation and relevance to the Ethiopian context. The pool of items drew upon diverse sources and viewpoints, anchoring the measurement approach in both empirical evidence and practical considerations from the field [45, 46]. This integrated process of systematically deriving items based on theory and formative research involving literature reviews, expert consultations, and stakeholder inputs helps establish content validity [26, 37].

### Language translation

The questionnaire was translated from English to Amharic using a forward translation process. Four independent translators, proficient in both Amharic and English, completed the forward translations. Subsequently, two additional language experts conducted back-translations of each forward translation into English. The back-translations were then compared with the English version to ensure the consistency of conceptual meaning and interpretive accuracy. This translation process comprehensively addressed translatability concerns before further refining the questionnaire through pilot testing and analysis. Following preliminary field testing and analysis, the translated version was empirically evaluated for reliability, validity, and cultural appropriateness within the target population and setting [38].

### Validity tests

Validity testing is crucial for ensuring that research tools accurately measure their intended constructs [47]. The development of reliable and valid tools involves several steps, including item generation, reliability assessment, and various forms of validity testing [48]. This process is particularly important in fields like health economics, where model validation tools can enhance the consistency and reproducibility of economic evaluations [49]. The need for robust tool development and validation extends to management sciences, where practitioners and theoreticians alike recognize the importance of developing standardized approaches for selecting appropriate research methods and techniques [50]. Such tools can improve the quality and reliability of research processes, addressing the challenges of creating and verifying new theories in management sciences. Face validity involved a panel of public health emergency management experts assessing the tool for relevance and clarity, resulting in refined item wording. Content validity was established using a Content Validity Index (CVI), which ensured all key aspects of public health emergency management were represented. This multi-faceted validation process guarantees that the tool accurately reflects critical constructs for reliable assessment and application.

### Face validity

A face validity study was conducted to evaluate the translated questionnaire prior to data collection. A total of 30 subject matter experts in PHEM working in the Ethiopia context were invited to participate. This number of experts falls within the recommended range of 25–75 respondents for face validity evaluation [51]. The questionnaire and study objectives were provided to the experts, with clear instructions to critically review each item. After one week, a panel discussion was held, during which the experts evaluated each item line-by-line and provided feedback and clarity/comprehension ratings on a 4-point scale.

The discussion focused on assessing whether the questionnaire items appropriately measured the research objectives in terms of relevance, representativeness, and comprehensiveness. All feedback was documented, and necessary revisions were made to the questionnaire. Items achieving a Face Validity Index (FVI) of at least 0.80 were retained in the final questionnaire. This 0.8 cutoff for the FVI was chosen based on widely accepted psychometric conventions and recommendations from the literature on instrument development and validation [37, 46, 52, 53]. Specifically, an FVI of 0.8 or higher is commonly considered an acceptable level indicating items are judged as clear and comprehensible by a sufficient proportion of expert raters [52, 53]. Using this 0.8 cutoff aligns with seminal guidance from Polit and Bec [37, 46]

Utilizing a 4-point relevance rating scale with multiple experts, as was done in the present study, adopts a conventional 0.8 standard that allows consistency with best practices, facilitates comparability to prior instrument validation studies across different fields, and represents an accepted balance between achieving adequate face validity while retaining sufficient content coverage[37].

Two methods were employed to determine the scale-level face validity index (S-FVI). The S-FVI/Ave took the mean of the Item-level Face Validity Index (I-FVI) scores across all items on the scale, alternatively calculated as the average clarity and comprehension proportions across raters. The S-FVI/UA represented the proportion of items that received full agreement (i.e., a rating of 3 or 4) from all raters. A universal agreement (UA) score of one was assigned if 100% agreement was achieved, otherwise 0. The S-FVI/UA was then calculated as the sum of UA scores divided by the total number of items.

In general, the I-FVI and the two S-FVI calculation methods—the average and universal agreement methods—provided quantitative indices to systematically evaluate face validity at both the item and scale levels during instrument development and validation.

### Content validity

Content validity of the questionnaire was established through an expert review involving eight PHEM experts with relevant educational qualifications. We conducted face validity assessments with experts to ensure that each item accurately reflected the constructs being measured. They independently rated each item on a 4-point Likert scale assessing how relevant the item was in measuring the designated construct. The scale was defined as: (1) irrelevant, (2) somewhat relevant, (3) quite relevant, and (4) highly relevant. Scores of 1–2 were coded as ‘0’ and 3–4 coded as ‘1’. After calculating Item-level content validity index (I-CVI), each item was judged as appropriate if the I-CVI was higher than 0.83 and eliminated if it was less than 0.83 [54, 55]. This cutoff value of 0.83 for an acceptable I-CVI was selected based on evidence-based recommendations from seminal content validity literature. With eight subject experts providing relevance ratings in the present study, Polit et al. [37] recommend a minimum I-CVI value of 0.83, demonstrating excellent content validity for an item to be retained. Utilizing this evidence-based 0.83 threshold follows validated guidelines and ensures only items achieving a sufficiently high degree of agreement among the expert panel regarding their relevance are included in the final instrument. A lower cutoff risked retaining items lacking adequate content validity support, while a higher value may have been overly stringent given the relatively small panel of 8 raters, potentially excluding too many items [54, 55].

### Factorial validity

Exploratory factor analysis (EFA) was selected for its strength in identifying underlying dimensions within complex constructs, making it especially suitable for capturing the multifaceted nature of PHEM [56]. This approach allowed us to empirically validate the tool’s structure by uncovering latent factors that represent critical components of emergency management, such as coordination, resource allocation, and system readiness. This expanded explanation underscores the relevance and rigor of our chosen methods, further enhancing the tool’s credibility and applicability in diverse settings. Prior to factor extraction, the Kaiser–Meyer–Olkin measure verified adequate sample size for a valid analysis [57, 58]. Initially, principal component analysis with Varimax rotation was performed to extract factors based on standard criteria, including eigenvalues exceeding 1 [29, 59], inspection of the Scree plot, and factor loadings above 0.4 [56, 60]. Further, parallel analysis was utilized as an empirical means of determining the optimal number of factors to retain, empirically accounting for inter-item correlations and strengthening validation of the revealed factor structure, following recommended statistical practices [29, 59, 60]. A minimum of 60% variance explained has been shown to provide good empirical support for confirming a measurement tool's construct validity, as referenced in seminal methodology texts [56].

### Internal consistency reliability test

The study used the pre-final version of the instrument to test the internal consistency reliability. Experts in selected national, regional, districts and facility participated, and their responses were analyzed to assess reliability.

### Participants and sample size

The study population consisted of public health emergency management (PHEM) experts working at national PHEM, across various regions, zones, and woredas (districts) of Ethiopia. The participants were selected from areas with different levels of experience in implementing PHEM programs, ensuring a diverse representation of perspectives and contexts.

During the tool development process, we conducted two regional consultation workshops: one in Adama during the desk review phase to gather stakeholder input and another in Hawassa-Sidama during instrument development to obtain feedback on the draft tool. For validity assessment, we purposively selected 30 face validity experts and 8 content validation experts from various regions, including Sidama, to ensure representation of diverse emergency management roles at both national and sub-national levels. This approach helped ensure that the content, structure, and questions of the tool were relevant and comprehensive.

For reliability testing, 260 professionals completed the survey instrument. These respondents were recruited across multiple regions in Ethiopia, with support from regional health bureaus, reflecting the intended national and regional user population involved in public health emergency management.

### Data collection procedure

The developed tool was distributed to the study sample for data collection. The tool was disseminated online via email using a secure survey platform. Participants received an email invitation with a brief introduction to the study aims and a link to access and complete the anonymous self-administered questionnaire electronically. This online distribution method enabled efficient data collection while adhering to social distancing protocols.

### Data analysis methods

The data analysis methods section outlines the steps taken to validate the psychometric properties of the developed tool through exploratory factor analysis for construct validity, Principal Component Analysis (PCA), internal consistency reliability testing using Cronbach's alpha, and Descriptive Statistics utilizing the Statistical Package for Social Sciences (SPSS) version 25 software. This provides a comprehensive overview of the data analysis methods used to ensure the tool accurately measures the intended constructs related to PHEM implementation status. It exhibits reliable and consistent measurement properties across diverse settings in Ethiopia, as follows.

### Exploratory factor analysis(EFA) and Principal component analysis(PCA)

Exploratory factor analysis (EFA) was employed to examine the underlying factorial structure of the questionnaire and validate its ability to measure the intended theoretical constructs consistently. The data's suitability for factor analysis was first assessed by examining the correlation matrix for coefficients exceeding 0.3, item communalities, and the results of Bartlett's Test of Sphericity and the Kaiser–Meyer–Olkin [61] Measure of Sampling Adequacy.

Principal Component Analysis (PCA) with Varimax rotation was then performed to extract factors, utilizing the criteria of eigenvalues greater than 1, inspection of the scree plot, and factor loadings above 0.4. Additionally, parallel analysis was conducted as an empirical method to determine the optimal number of factors to retain, accounting for inter-item correlations.

### Internal consistency reliability

The internal consistency reliability of the instrument was evaluated using Cronbach's alpha. This statistic measures the homogeneity of items within each scale, with values of 0.70 or higher indicating adequate internal consistency [62].

### Descriptive statistics

Descriptive statistics, including frequencies and percentages, were calculated to summarize the socio-demographic characteristics of the study participants.

### Ethical clearance

Ethical approval for this tool development and validation study was granted by the Institutional Review Board of the Ethiopian Public Health Institute (EPHI IRB) under reference number EPHI 6.13/68 on 19 July 2023. All participants provided informed consent after being informed about the study objectives to develop and validate an assessment tool and the procedures, potential risks, and benefits of participation. They were assured of the confidentiality of their data and responses, their right to withdraw at any time without reprisal, and that anonymity would be maintained.

## Results

### Socio-demographic characteristics of participants

A study was conducted with 260 PHEM experts, achieving a 100% response rate with no missing data. More than two-thirds of participants were male (73.9%) (Table 1). Nearly half of the participants (45.4%) were between 20 and 30 years of age, indicating a comparatively youthful demographic that contributes to the overall diversity and energy of the group, while also raising questions about the levels of experience. Regarding educational status, 53.85% of participants held a first degree (bachelor’s), amounting to 140 individuals, while 46.15% held a master’s degree, which corresponds to 120 participants. Half of the experts worked in health facilities, and the majority (50.4%) were based in Addis Ababa.

**Table 1:** Socio-demographic profile of the PHEM experts participated in the pilot survey (n = 260) in Ethiopia

Variables	Frequency	Percentage (%)
Age		
20–30	118	45.38
31–40	113	43.46
41–50	26	10.00
51–60	3	1.15
Sex		
Male	192	73.85
Female	68	26.15
Level of education		
First degree/bachelor’s	140	53.85
Master’s degree	120	46.15
Years of experience		
< 5	49	18.92
5–10	115	44.40
> 10	95	36.68
Facility type		
Health facility	130	50.00
District PHEM office	62	23.85
Zonal PHEM office	34	13.08
Regional PHEM office	24	9.23
University	8	3.08
Other sectors	2	0.77
Region		
Addis Ababa	131	50.38
Afar	24	9.23
Sidama	67	25.77
South-west	38	14.62

### Identification of content domains and item generation

A total of 60 items were carefully generated to reflect these conceptual domains based on empirical evidence and stakeholder consultation, ensuring content representation and local relevance. Specifically, 15 items addressed overall implementation status, 15 evaluated individual-level capacities and attitudes, and 30 assessed organizational characteristics (Additional file 1).

### Forward and backward translation

The questionnaire was initially developed in English. It was then translated from English into Amharic. A comparison of the back-translation from Amharic into English with the original English questionnaire found no discrepancies, indicating no modifications were required. Similarly, there were also no adjustments needed when evaluating consistency between the back-translated version into English and original English questionnaire.

### Content evidence of validity

**Fig. 1:**
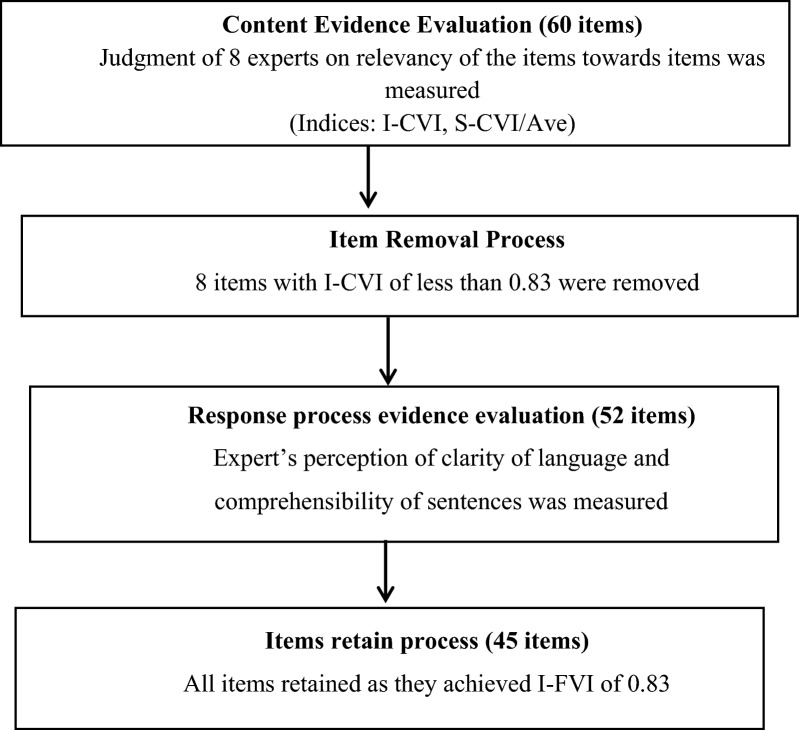
Flowchart of the validation of process

The original questionnaire consisted of 60 items across domains. I-CVI values revealed eight items had inadequate relevance with scores below the predefined threshold of 0.83. These items were excluded to improve content validity (Fig. 1). Re-computation of S-CVI using the Average Method (S-CVI/Ave), which measures the scale's content validity by averaging I-CVIs, confirmed enhanced content validity of the remaining 52 items with S-CVI/Ave exceeding 0.83 for all factors.

### Response process validity

The face validity of the translated questionnaire was assessed through expert evaluation by 30 Ethiopian PHEM professionals with at least a master's degree and over five years of experience. The experts rated each item on a 4-point Likert scale to assess clarity and comprehensibility. Item-level Validity Index (I-VI) and Scale-level Validity Index (S-VI) were calculated based on the ratings. Additionally, the average proportion of items judged as clear/comprehensible by all raters was computed (Table 2).

**Table 2:** Validation of the content and face validity of the comprehensive public health emergency management program implementation assessment tool

Items	Content validity	Response process validity	
	S-CVI/Ave (60 items)	S-CVI/Ave (52 items)	S-FVI/Ave (52 items)
PHEM Program Implementation Status Assessment	0.91	0.92	0.85
Individual characteristics			
Capacity building	0.91	0.94	0.97
Availability of reinforcement	0.81	0.94	0.93
Engagement	0.94	0.94	0.95
Attitude towards PHEM	1	1	0.92
Work related factors	0.9	1	0.93
Organizational factors			
Information sharing	0.88	0.94	0.87
Integration	0.88	0.88	0.92
Planning	0.88	0.94	0.88
Delegation	0.82	0.88	0.97
Availability of resource and funding	0.8	0.88	0.96
Leadership	0.94	0.94	0.98
Effective monitoring and evaluation	0.88	0.88	0.98
Coordination and collaboration	0.88	0.88	0.93
Infrastructure	0.88	0.88	0.97
Legal framework	0.85	0.91	0.87

The Item Face Validity Index (I-FVI) scores, demonstrating clarity of individual items, ranged from 0.85 to 0.98. The Scale Face Validity Index (S-FVI) based on universal agreement for each of the 15 dimensions and the overall Scale Content Validity Index average (S-CVI/Ave) across all 52 items were also above the predefined acceptable level of 0.8 (Additional file 2)

### Factor analysis: testing validity of the instrument

**Fig. 2:**
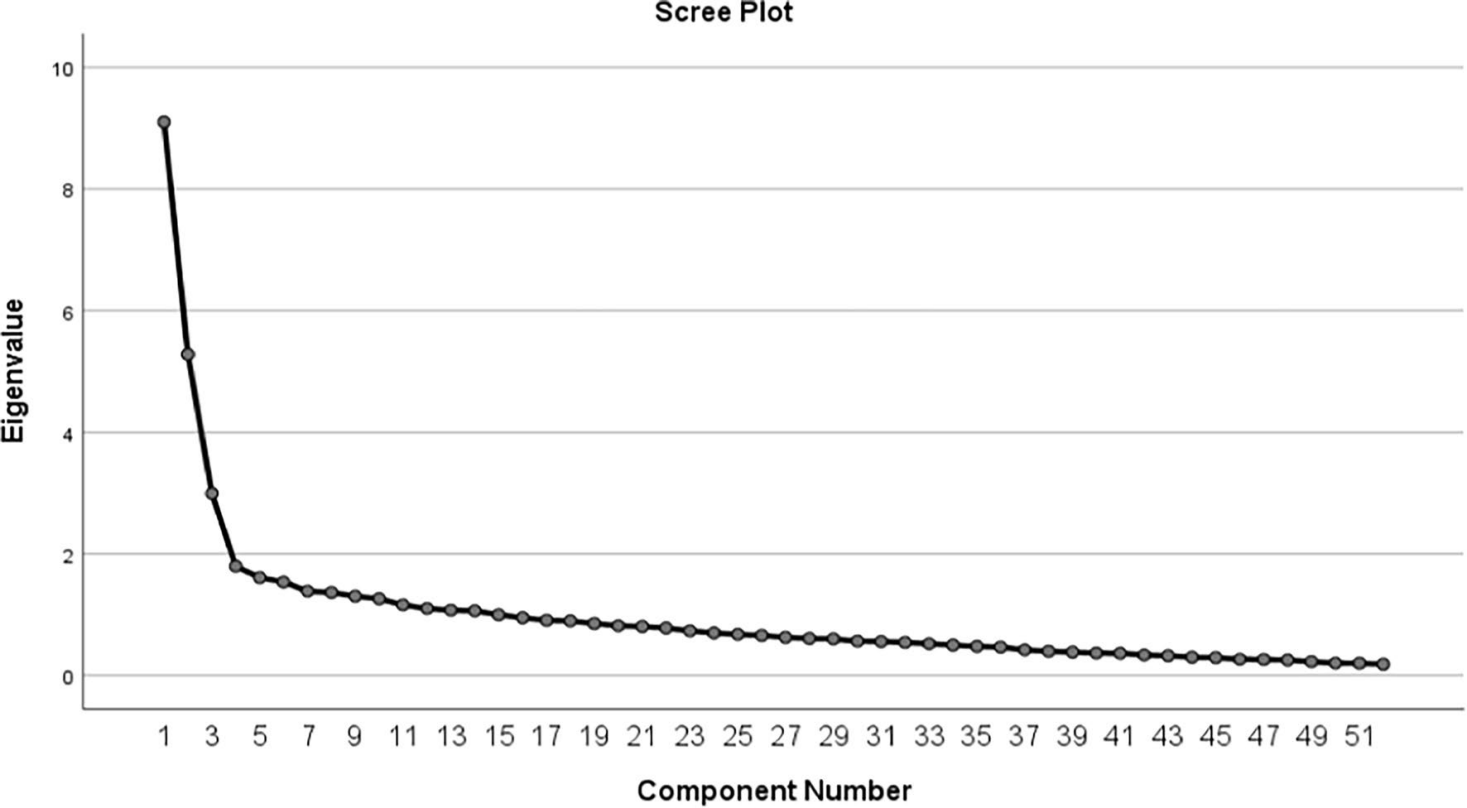
Scree plot for the extracted items

The suitability of the data for factor analysis was first evaluated. The correlation matrix showed many coefficients exceeding 0.3, while item communalities ranged from 0.526 to 0.808, indicating acceptable variance explanation. Bartlett's Test of Sphericity was highly significant, confirming suitable inter-item correlations. The Kaiser–Meyer–Olkin Measure of Sampling Adequacy was 0.831, above the 0.800 threshold for good factorability (Fig. 2).

Initial factor extraction identified 16 factors accounting for 65.31% of the data variance. Screen plot visualization confirmed retention of 16 factors. The initial EFA revealed that two items related to PHEM implementation status ("PHEM1"and"PHEM2") did not load significantly onto any dimension. Therefore, these items were removed from further analysis (Table 3).

**Table 3:** Exploratory Factor Analysis Results of the PHEM Implementation Status Assessment Tool

Items	1	2	3	4	4	5	6	7	8	9	10	11	12	13	14
PHEM implementation status															
PHEM 3	0.568														
PHEM 4	0.662														
PHEM 5	0.621														
PHEM 6	0.631														
PHEM 7	0.524														
PHEM 8	0.547														
PHEM 9	0.633														
PHEM 10	0.652														
PHEM 11	0.705														
PHEM 12	0.694														
PHEM 13	0.709														
PHEM 14	0.674														
Individual level factors															
Capacity building															
ICapacity1		0.722													
ICapacity2		0.662													
ICapacity3		0.765													
Availability of reinforcement															
IAvalibility1			0.761												
IAvalibility2			0.779												
Engagement															
Iengagement1				0.760											
Iengagement2				0.747											
Iengagement3				0.693											
Iengagement4				0.691											
Attitude towards PHEM															
Iattitude1					0.644										
Iattitude2					0.834										
Organizational level factors															
Information sharing															
Cinformation1						0.614									
Integration															
Cintegration1							0.663								
Cintegration2							0.768								
Planning															
Cplanning1								0.589							
Cplanning2								0.755							
Delegation															
Cdelegation1									0.783						
Cdelegation2									0.800						
Availability of resources and funding															
Cavailability1										0.825					
Cavailability2										0.791					
Leadership															
Ileadership1											0.852				
Ileadership2											0.730				
Effective monitoring and evaluation															
CME1												0.717			
CME2												0.849			
CME3												0.758			
Infrastructure															
Cinfrastructure1													0.865		
Cinfrastructure2													0.863		
Coordination and collaboration															
Ccoordination1														0.797	
Ccoordination2														0.779	
Legal framework															
Clegal1															0.574
Clegal2															0.669
Clegal3															0.786
Clegal4															0.716

A second EFA was then conducted without the two previously removed items. The results identified additional items that did not adequately fit any factors, including two related to work factors ("Iworkrelated1"and"Iworkrelated2"), two on resource availability ("Cavailability3"and"Cavailability4") and one on information sharing ("Cinformation2"). After removing these items, the analysis confirmed a 15-dimensional structure with 45 items (Additional file 3) aligning with the theoretical framework. The Kaiser–Meyer–Olkin measure of sampling adequacy was 0.840, indicating an appropriate sample size. The identified factors were: PHEM implementation status (items PHEM3-14), individual capacity building (ICapacity1 to ICapacity3), individual reinforcement availability (Iavailability1-2), individual engagement (Iengagement1-4), individual attitude (Iattitude1-2), community information sharing (Cinformation1), community integration (Cintegration1-2), community planning (Cplanning1-2), community delegation (Cdelegation1-2), community resource availability (Cavailability1-2), community leadership (Cleadership1-2), community M&E (CME1-2), community coordination (Ccoordination1-2), community infrastructure (Cinfrastructure1-2), and community legal framework (Clegal1-4). The fifteen dimensions explained a total of 74.801 per cent of the variance among items in the study. Bartlett's Test of sphericity proved significant, and all communalities were over the required value of 0.500 (Table 4).

**Table 4:** The Eigenvalues and total variance explained by the extracted items among PHEM experts, Ethiopia, 2024 (n = 260)

Factor	Initial Eigen Values			Extraction sums of squared loadings			Rotation Sums of Squared Loadings		
	Total	% of Variance	Cumulative %	Total	% of Variance	Cumulative %	Total	% of Variance	Cumulative %
1	7.575	25.251	25.251	7.575	25.251	25.251	2.352	7.840	7.840
2	2.538	8.460	33.712	2.538	8.460	33.712	2.324	7.739	9.013
3	1.605	5.350	39.061	1.605	5.350	39.061	2.301	7.691	11.342
4	1.479	4.930	43.991	1.479	4.930	43.991	2.292	7.640	15.481
5	1.392	4.640	48.631	1.392	4.640	48.631	2.262	7.539	23.020
6	1.208	4.025	52.657	1.208	4.025	52.657	1.872	6.241	29.261
7	1.125	3.749	56.406	1.125	3.749	56.406	1.736	5.785	35.046
8	1.091	3.636	60.041	1.091	3.636	60.041	1.689	5.631	40.677
9	1.023	3.411	63.452	1.023	3.411	63.452	1.671	5.571	46.247
10	1.392	3.089	66.541	1.392	3.089	66.541	1.616	5.386	51.634
11	1.208	3.022	69.563	1.208	3.022	69.563	1.592	5.308	56.941
12	0.998	2.954	70.142	0.998	2.954	70.142	1.560	5.202	62.143
13	0.948	2.732	71.464	0.948	2.732	71.464	1.367	4.556	66.699
14	0.927	2.676	72.239	0.927	2.676	72.239	1.260	4.202	70.901
15	0.907	2.562	74.801	0.907	2.562	74.801	1.170	3.900	74.801

### Internal reliability of the instrument

The internal consistency reliability of the 45-item instrument was assessed using Cronbach's alpha, which evaluates the homogeneity of items within a scale to indicate how well they measure the same underlying construct. The instrument demonstrated excellent internal consistency, with the Cronbach's alpha coefficient for the overall scale being 0.863, well above the acceptable threshold. All subscales also exhibited adequate internal consistency based on commonly accepted reliability standards. Cronbach’s α was used to measure the internal consistency of scale items, and for the whole scale, the Cronbach’s α value was 0.863, demonstrating very good reliability (Table 5).

**Table 5:** Internal consistency reliability measured by cronbach's alpha for the factorially derived domains/scales

Item	Scale mean if item deleted	Scale variance if item detected	Corrected item-total correlation	Squared multiple correlation	Cronbach’s alpha if item deleted
PHEM implementation status					
PHEM 3	116.17	520.854	0.229	0.357	0.863
PHEM 4	116.29	521.768	0.210	0.431	0.863
PHEM 5	116.33	524.571	0.166	0.432	0.864
PHEM 6	116.32	527.639	0.120	0.440	0.865
PHEM 7	116.17	527.639	0.136	0.360	0.864
PHEM 8	116.27	519.489	0.237	0.343	0.863
PHEM 9	116.13	522.791	0.183	0.415	0.864
PHEM 10	116.05	527.727	0.135	0.285	0.864
PHEM 11	116.39	520.432	0.211	0.493	0.863
PHEM 12	116.88	523.330	0.161	0.567	0.864
PHEM 13	116.72	522.768	0.179	0.559	0.864
PHEM 14	116.54	519.616	0.233	0.489	0.863
Individual level factors					
Capacity building					
ICapacity1	117.21	511.743	0.373	0.318	0.860
ICapacity2	116.79	518.173	0.272	0.297	0.862
ICapacity3	117.10	518.036	0.288	0.327	0.862
Availability of reinforcement					
IAvalibility1	116.72	513.201	0.352	0.395	0.860
IAvalibility2	117.03	510.254	0.398	0.377	0.859
Engagement					
Iengagement1	117.00	507.371	0.422	0.416	0.859
Iengagement2	117.36	514.825	0.366	0.366	0.860
Iengagement3	117.23	511.640	0.364	0.464	0.860
Iengagement4	117.21	513.146	0.378	0.377	0.860
Attitude towards PHEM					
Iattitude1	117.37	508.536	0.434	0.366	0.859
Iattitude2	116.38	517.960	0.290	0.328	0.861
Community level factors					
Information sharing					
Cinformation1	117.38	512.616	0.345	0.489	0.860
Integration					
Cintegration1	117.21	511.493	0.382	0.564	0.860
Cintegration2	117.25	509.323	0.432	0.470	0.859
Planning					
Cplanning1	117.21	506.304	0.452	0.443	0.858
Cplanning2	117.13	510.611	0.436	0.354	0.859
Delegation					
Cdelegation1	117.33	508.823	0.472	0.507	0.858
Cdelegation2	117.18	513.330	0.379	0.438	0.860
Availability of resources and funding					
Cavailability1	116.59	518.289	0.258	0.491	0.862
Cavailability2	116.99	514.540	0.369	0.504	0.860
Leadership					
Ileadership1	117.12	507.384	0.479	0.512	0.858
Ileadership2	117.07	506.350	0.485	0.531	0.858
Effective monitoring and evaluation					
CME1	117.35	508.808	0.440	0.553	0.859
CME2	117.33	512.455	0.385	0.606	0.860
CME3	117.32	509.339	0.442	0.608	0.859
Infrastructure					
Cinfrastructure1	117.08	515.369	0.320	0.627	0.861
Cinfrastructure2	117.14	516.498	0.320	0.614	0.861
Coordination and collaboration					
Ccoordination1	117.31	511.812	0.412	0.477	0.859
Ccoordination2	117.13	513.852	0.410	0.394	0.859
Legal framework					
Clegal1	117.27	509.632	0.452	0.519	0.859
Clegal2	117.20	514.645	0.385	0.506	0.860
Clegal3	117.18	520.419	0.298	0.464	0.861
Clegal4	117.28	516.133	0.363	0.378	0.860

## Discussion

This study addresses a critical gap in public health emergency management (PHEM) by developing and validating the first standardized, psychometrically robust tool to assess implementation status comprehensively. Employing a mixed-methods approach, we integrated literature reviews, stakeholder consultations, and rigorous psychometric validation procedures to ensure the tool's relevance and applicability. The final 45-item instrument demonstrated strong psychometric properties, achieving high content validity (CVIs > 0.83), robust construct validity with a 15-factor structure explaining 74.8% of variance, and excellent reliability (overall α = 0.863, subscales > 0.70). In alignment with our findings, previous studies have identified gaps in existing public health emergency management tools, often focusing narrowly on specific aspects of response. Compared to previous studies, our tool provides a more integrated approach by addressing both structural and procedural aspects of PHEM. This holistic perspective allows for a more comprehensive assessment of emergency preparedness, ultimately leading to more effective interventions.

In alignment with Donabedian's Structure-Process-Outcome Model, our tool emphasizes critical determinants of effective emergency management. The structural components include resources and workforce capacity, while the process elements focus on multi-sectoral coordination and resource allocation. By systematically assessing these domains, the tool not only measures the implementation status of PHEM programs but also elucidates how improvements in these areas can lead to enhanced public health outcomes and community resilience.

The implications of these findings are profound; they suggest that targeted interventions in the identified domains could significantly bolster emergency preparedness capabilities. Additionally, the tool's robust psychometric foundation positions it as a replicable model for other nations, facilitating the development of locally tailored PHEM assessments. Ultimately, by integrating rigorous statistical validation with established theoretical frameworks, this study advances the discourse on emergency preparedness, highlighting the necessity for evidence-based tools that enhance public health outcomes globally.

This assessment assessment tool, while developed with Ethiopian contexts in mind, holds significant global relevance and adaptability for low-resource settings worldwide. Its structured approach to evaluating public health emergency management can be tailored to meet the unique needs and challenges of various regions, such as those facing similar health crises due to climate change, urbanization, or conflict. By incorporating local language translations, cultural considerations, and specific health threats relevant to different countries, the tool can facilitate comprehensive assessments in diverse environments. Additionally, its focus on multi-sectoral coordination and resource allocation is universally applicable, enabling health authorities globally to identify gaps, enhance preparedness, and foster resilience in the face of public health emergencies. This adaptability ensures that the tool can serve as a valuable resource for countries in need of robust emergency management frameworks, ultimately contributing to improved health outcomes and community resilience on an international scale.

Our evaluation of item and scale relevance through I-CVI and S-CVI/Ave scoring confirmed that all aspects exceeded the predefined thresholds of 0.83, validating the tool's accuracy in measuring the intended constructs. This result is consistent with existing literature advocating for content validity assessments via expert review and scoring [8, 54]. In contrast, prior studies often lacked rigorous validation processes, leading to inconsistent or incomplete results that failed to adequately capture program implementation status [10, 13, 63, 64]. Our findings also indicate that face validity testing demonstrated strong evidence of readability and comprehensibility, with I-FVI and S-FVI indices for expert ratings on item clarity surpassing the cut-off of 0.8. This confirms the questionnaire's clarity, aligning with literature that supports using I-FVI and S-FVI indices for validating face validity [26, 28, 51]. Many earlier studies relied solely on qualitative methods, lacking quantitative face validity assessment [12, 13].

Furthermore, our factorial validity analysis, conducted through exploratory factor analysis, revealed a theoretically aligned 15-dimensional structure that explained over 74.8% of variance among items. This method adheres to the recommended practices for validating construct validity, offering a more rigorous validation compared to exploratory analyses used in some prior research [12, 13]. Additionally, we found a high level of internal consistency reliability, with a Cronbach's alpha of 0.863, significantly above the common standard of 0.7. This strong evidence indicates that the items reliably measure the same underlying constructs, surpassing recognized reliability benchmarks [25, 56, 62, 63]. In contrast, several past qualitative studies did not quantify reliability [12, 13].

The developed and validated 45-item tool offers a comprehensive approach for assessing public health emergency management (PHEM) implementation status across diverse domains [29, 33]. Developed countries with well-established PHEM programs can leverage this tool to identify potential gaps, benchmark their progress, and prioritize areas for further enhancement [5, 12]. By evaluating critical aspects such as multi-sectoral coordination, resource allocation, transparency, workforce capacity, and provision of essential supplies, the tool enables developed nations to maintain a high level of emergency preparedness and response capabilities [12, 16]. Furthermore, the tool's strong psychometric properties, as shown above, ensure that the assessment results are reliable and accurately reflect the current implementation status [37, 46, 51–54, 59, 60].

While this tool was developed and validated specifically for the Ethiopian context, the methodological approach employed in this study, including the use of psychometric methods for instrument development and validation, offers a guiding framework for other nations, particularly those with limited resources, to develop their own contextually tailored instruments for assessing public health emergency management knowledge, attitudes, and practices. Each nation would need to undertake a similar process of adapting the tool to align with their specific context, local terminology, and priority competency areas in PHEM [8]. These nations often face infrastructural limitations, workforce shortages, and funding constraints in implementing effective PHEM programs [4, 8–10]. Low-resource countries frequently struggle with barriers like inadequate healthcare workforces, deficient surveillance systems, and lack of inter-sectoral collaboration factors, that can undermine emergency preparedness and response efforts [13, 63, 65, 66]. Nonetheless, the comprehensive nature of the tool allows for a holistic assessment of PHEM readiness, considering factors like workforce capacity, resource availability, and multi-sectoral collaboration areas that are commonly identified as deficient in developing countries [13, 63]. However, practical challenges such as accessibility to remote areas, limited resources for data collection, and lack of trained personnel may hinder the tool's widespread implementation in these settings [67]. Additionally, cultural and linguistic adaptations for local contexts may be necessary to ensure the tool's relevance, comprehensibility, and acceptance in diverse settings within these countries [38]. Engaging local stakeholders and adapting the tool through rigorous translation and contextualization processes can enhance its applicability and uptake in resource-limited environments [68].

Upon reviewing the results of our content validity assessment during tool development, several key challenges emerged that provide valuable insights for strengthening future validation approaches in a cohesive manner. Consistent with established guidance [46, 69], items receiving an I-CVI below 0.83 for relevance were removed to uphold standards of content validity, however, solely relying on quantitative metrics may have overlooked nuanced qualitative perspective important for complex borderline cases [55, 70], as some studies note that while quality thresholds facilitate objective review, they risk ignoring valuable debate when multi-dimensional evaluation of conceptually complex items is required [55, 70], which resonated with our experience as experts understandably differed on specific items given their subjective nature. While scores structured appraisal [37, 55, 69], transparently documenting these discussions also emerged as an important lesson learned [37, 55, 69]. More transparent documentation of these discussions, including rationales for divergent viewpoints and consensus building, may have better captured these nuances beyond summary outcomes alone [37, 71]. An overarching lesson is the need for flexibility and integration within the process to respect multiple valid viewpoints, especially for complex cases challenging simple resolution [37, 46, 69, 70, 72]. Prioritizing open dialogue and customized handling of differing opinions, over rigid adherence to scores or timelines, more fully addresses construct validity [37, 70, 72, 73]. Hence, prioritizing numerical cutoffs over flexible consideration of multiple valid viewpoints could compromise construct validity attainment [70, 71]. Our results hint at tensions between quantitative and qualitative judgment that call for processes respecting both empirical evidence and practical multi-faceted feedback integration. Reflecting on challenges faced informs opportunities to strengthen validation rigor moving forward in future efforts which may benefit from a balanced mixed-methods approach, combining metrics with documenting differing rationales to respect diverse stakeholder stances during scale refinement [37, 55, 69]. Taking a balanced, evidence-based approach combining quantitative metrics with subjective domain knowledge will help generate valid tools grounded in empirical evidence as well as practical perspectives [37, 46, 69, 70, 72, 73].

The development and validation of this tool have significant implications for future research and implementation efforts. Further research could focus on adapting and validating the tool for specific cultural or linguistic contexts within developing countries, enhancing its applicability and acceptability in diverse settings [38]. Implementation studies could examine the tool's effectiveness in guiding PHEM program improvements, resource allocation, and capacity-building efforts in resource-constrained settings [12, 13, 63]. Longitudinal studies could assess the tool's responsiveness in capturing changes in PHEM implementation status over time, informing program monitoring and evaluation efforts [12]. Additionally, research on integrating the tool into existing PHEM frameworks and policies, as well as revising the tool through time as needed, could facilitate its widespread adoption and institutionalization at various levels, from local to national [8].

While this study offers valuable insights into public health emergency management (PHEM) in Ethiopia, the findings also hold significant potential for application in other low- and middle-income settings with appropriate adaptations. The comprehensive nature of the 45-item tool, validated through rigorous psychometric methods, allows for flexibility in addressing the unique challenges faced by different contexts. By leveraging established frameworks, such as Donabedian's Structure-Process-Outcome Model, we can adapt the tool to account for variations in resource allocation, multi-sectoral coordination, and workforce capacity across diverse settings. For instance, in low-resource settings, the tool can be applied to assess coordination efforts during a health crisis, illustrating its practical utility.

This adaptability ensures that the tool can effectively guide public health practitioners in assessing and improving emergency readiness, regardless of the specific socio-economic or infrastructural conditions present. The assessment tool can be tailored to various global contexts, such as conflict zones and areas affected by climate change, enabling it to address diverse public health challenges. Moreover, engaging with stakeholders from various low- and middle-income countries will facilitate the contextualization of the tool, allowing for localized modifications that enhance its relevance and effectiveness. By framing our findings within this broader context, we aim to contribute to a more robust understanding of how structured assessments can lead to improved health outcomes and community resilience in emergency situations globally.

### Strengths and limitations

The study applied rigorous validation methods including I-CVI/S-CVI scoring, I-FVI/S-FVI indices, factor analysis, and reliability testing to validate the tool. This was the first tool to undergo such extensive psychometric evaluation for systematically measuring Ethiopia's PHEM programs.

Validations were conducted in a single country context, so generalizability to other settings requires further testing. Pilot testing enrolled experts from major regions, but additional evaluation in varied field contexts may provide further insights. Moreover, a significant number of participants (45.4%) were aged 20–30, which should be considered when interpreting the findings. This age distribution may raise questions about the depth of experience among those regarded as experts, as expertise encompasses not only years of work but also relevant training and skills. Additionally, potential disparities between different area types were not explicitly discussed, which may influence the applicability of the findings across diverse contexts. However, since we have involved experts from various regions, this may not be a major problem in interpreting the current findings. We also acknowledge potential biases from stakeholder input and the need for cultural adaptation in diverse contexts.

### Recommendations

Based on our study’s findings, we present actionable policy recommendations to enhance public health emergency management (PHEM) readiness. First, we recommend operationalizing the validated PHEM tool within national public health programs and emergency preparedness plans, establishing clear implementation guidelines to ensure consistent application and data-driven decision-making across various contexts. Second, incorporating theoretical models such as the PRECEDE-PROCEED model can address key predisposing, enabling, and reinforcing factors that influence public health emergency readiness, thereby linking the tool to specific programmatic improvements and measurable outcomes. Third, we emphasize the importance of demonstrating practical applications by illustrating how the tool can impact critical areas of public health intervention, including governance, workforce capacity, and resource allocation. Policymakers should prioritize resource investments informed by the tool’s findings, focusing on essential medical supplies and infrastructure improvements to strengthen structural metrics for PHEM readiness. Leveraging the tool for ongoing monitoring and evaluation will enable systematic tracking of progress, benchmarking, and identification of gaps, guiding targeted resource allocation. Furthermore, reinforcing leadership engagement and community participation can drive tailored programming and capacity-building initiatives, contributing to long-term improvements in emergency readiness. These recommendations aim to maximize the tool’s relevance and effectiveness in enhancing public health outcomes. While the PHEM assessment tool was developed with the Ethiopian context in mind, it possesses significant global adaptability and relevance for low-resource settings worldwide. Its structured evaluation approach can be tailored to address the unique challenges and needs of diverse regions experiencing health crises driven by climate change, urbanization, or conflict. By incorporating local language translations, cultural adaptations, and health threat-specific considerations, the tool can facilitate comprehensive assessments across varied contexts. Its emphasis on multi-sectoral coordination and strategic resource allocation is broadly applicable, enabling health authorities globally to identify preparedness gaps, enhance emergency readiness, and build resilience against public health crises. This adaptability ensures that the tool serves as a valuable resource for countries in need of robust emergency management frameworks, ultimately contributing to improved health outcomes and strengthened community resilience on an international scale.

## Conclusions

This study successfully develops and validates a comprehensive tool for assessing public health emergency management (PHEM) readiness, demonstrating strong psychometric properties, including high content validity, robust construct validity, and excellent reliability. The incorporation of Donabedian's Structure-Process-Outcome model enhances the study's scientific rigor and applicability. By framing the tool within this theoretical context, we can clearly link validated components to real-world outcomes. The tool effectively measures critical structural components such as resource availability and workforce capacity, essential for effective emergency management. Additionally, it provides valuable insights into crucial processes like multi-sectoral coordination and transparency, highlighting their importance in strengthening emergency preparedness. By enhancing structural and process elements, we anticipate improved outcomes as per the Donabedian framework. Although the study does not directly measure outcomes, both frameworks allow for predictions regarding improvements in public health resilience and emergency readiness. Overall, this integration of public health models elevates the study from a descriptive validation exercise to a comprehensive tool capable of driving systemic improvements in public health emergency management. By establishing these connections, the tool can inform strategic policy decisions and enhance emergency readiness in both Ethiopia and other low-resource settings.

## Supplementary Information

Additional file 1.

## Abbreviations

CVI: Content validity index
EME: Effective monitoring and evaluation
EFA: Exploratory factor analysis
FVI: Face validity index
I-CVIs: Item-level content validity index
I-FVI: Item-level face validity index
I-VI: Item-level validity index
KMO: Kaiser-Meyer-Olkin
PHEM: Public health emergency management
PCA: Principal component analysis
S-CVI: Scale-level content validity index
S-CVI/Ave: Scale-level content validity index based on the average method
S-FVI/Ave: Scale-level face validity index based on the average method
S-FVI/UA: Scale-level face validity index based on the universal agreement method
S-VI: Scale-level validity index
UA: Universal agreement

## References

[1] Guha-Sapir D, Below R, Hoyois P, EM-DAT: the CRED/OFDA international disaster database. 2016

[2] Örgütü DSJE, WHO coronavirus (COVID-19) dashboard. 2021

[3] Bell AJ et al., Disease control and Ebola in West Africa: a qualitative evidence synthesis. 2022; 4(1)

[4] Otto IM, et al. Social vulnerability to climate change: a review of concepts and evidence. Regional Environ Change. 2017;17:1651–62.

[5] Kruk ME, et al. Mortality due to low-quality health systems in the universal health coverage era: a systematic analysis of amenable deaths in 137 countries. The Lancet. 2018;392(10160):2203–12. 10.1016/S0140-6736(18)31668-4 PMC6238021 30195398

[6] SDGs UJRJ, UN Sustainable Development Goals, Knowledge Platform. 2015; 26: 2019

[7] Terwee CB, et al. Quality criteria were proposed for measurement properties of health status questionnaires. J Clin Epidemiol. 2007;60(1):34–42. 17161752 10.1016/j.jclinepi.2006.03.012

[8] Polit DF, Beck CT. Generalization in quantitative and qualitative research: Myths and strategies. Int J Nurs Stud. 2010;47(11):1451–8. 20598692 10.1016/j.ijnurstu.2010.06.004

[9] Keszei AP, Novak M, Streiner DL. Introduction to health measurement scales. J Psychosomatic Res. 2010;68(4):319–23. 10.1016/j.jpsychores.2010.01.006 20307697

[10] Board GPM, A world in disorder: Global Preparedness Monitoring Board annual report 2020. 2020

[11] Garg R, Bhargava A, Singh SK. Capacity building in public health emergency management: A crucial pillar for global health security. NMO J. 2024;18(1):28–32.

[12] Rose DA, et al. The evolution of public health emergency management as a field of practice. Am J Public Health. 2017;107(S2):S126-s133. 10.2105/ajph.2017.303947. 28892444 10.2105/AJPH.2017.303947 PMC5594387

[13] Mandyata CB, Olowski LK, Mutale W. Challenges of implementing the integrated disease surveillance and response strategy in Zambia: a health worker perspective. BMC Public Health. 2017;17(1):1–12. 28950834 10.1186/s12889-017-4791-9 PMC5615443

[14] World Health Organization, A strategic framework for emergency preparedness. 2017

[15] Mandyata CB, Olowski LK, Mutale W. Challenges of implementing the integrated disease surveillance and response strategy in Zambia: a health worker perspective. BMC Public Health. 2017;17(1):746. 10.1186/s12889-017-4791-9. 28950834 10.1186/s12889-017-4791-9 PMC5615443

[16] Adane A, et al. Routine health management information system data in Ethiopia: consistency, trends, and challenges. Glob Health Action. 2021;14(1):1868961. 10.1080/16549716.2020.1868961. 33446081 10.1080/16549716.2020.1868961 PMC7833046

[17] Alemu T, et al. Evaluation of public health surveillance system performance in Dangila district, Northwest Ethiopia: a concurrent embedded mixed quantitative/qualitative facility-based cross-sectional study. BMC Public Health. 2019;19(1):1343. 10.1186/s12889-019-7724-y. 31640662 10.1186/s12889-019-7724-y PMC6805593

[18] van den Berg H, et al. Perceived needs of disease vector control programs: a review and synthesis of (sub) national assessments from South Asia and the Middle East. PLoS Negl Trop Dis. 2024;18(4):e0011451. 38630832 10.1371/journal.pntd.0011451 PMC11075900

[19] Lee AC, et al. The state of integrated disease surveillance globally: synthesis report of a mixed methods study. Public Health. 2024;228:85–91. 38340506 10.1016/j.puhe.2024.01.003

[20] Ruckert A, et al. One Health governance principles for AMR surveillance: a scoping review and conceptual framework. Public Health. 2024;2:e4.

[21] Scanlon M, Taylor E, Waltz K. Evaluating efficacy of a COVID-19 alternative care site preparedness assessment tool for catastrophic healthcare surge capacity during pandemic response. Healthcare. 2023;11(3):324. 36766899 10.3390/healthcare11030324 PMC9914666

[22] Elhakim M, et al. Learning interventions in the WHO Eastern Mediterranean region: supporting Member States to get prepared for better response to health emergencies in the region. Front Public Health. 2024;12:1441223. 10.3389/fpubh.2024.1441223. 39329002 10.3389/fpubh.2024.1441223 PMC11424434

[23] Donabedian A. Evaluating the quality of medical care. Milbank Memorial Fund Quarter. 1966;44(3):166–206. 5338568

[24] Arah OA, et al. A conceptual framework for the OECD health care quality indicators project. Int J Q Health Care. 2006;18(1):5–13. 10.1093/intqhc/mzl024 16954510

[25] Anthoine E, et al. Sample size used to validate a scale: a review of publications on newly-developed patient reported outcomes measures. Health Q Life Outcomes. 2014;12(1):1–10. 10.1186/s12955-014-0176-2 PMC4275948 25492701

[26] Zamanzadeh V, et al. Design and implementation content validity study: development of an instrument for measuring patient-centered communication. J Caring Sci. 2015;4(2):165. 26161370 10.15171/jcs.2015.017 PMC4484991

[27] Downe-Wamboldt B. Content analysis: method, applications, and issues. Health Care Women Int. 1992;13(3):313–21. 1399871 10.1080/07399339209516006

[28] de Souza AC, Alexandre NMC, Guirardello E. Psychometric properties in instruments evaluation of reliability and validity. Epidemiologia e Serviços de Saúde. 2017;26:649–59. 28977189 10.5123/S1679-49742017000300022

[29] Streiner DL, Kottner J. Recommendations for reporting the results of studies of instrument and scale development and testing. J Adv Nurs. 2014;70(9):1970–9. 24684713 10.1111/jan.12402

[30] Artino Jr AR et al., *Developing questionnaires for educational research: AMEE Guide No. 87.* 2014; 36(6): 463–474 10.3109/0142159X.2014.889814 PMC4059192 24661014

[31] Boateng GO, et al. Best practices for developing and validating scales for health, social, and behavioral research: a primer. Front Public Health. 2018;6:149. 29942800 10.3389/fpubh.2018.00149 PMC6004510

[32] Saunders B, et al. Saturation in qualitative research: exploring its conceptualization and operationalization. Q Quant. 2018;52:1893–907. 10.1007/s11135-017-0574-8 PMC5993836 29937585

[33] Ayre C, et al. Critical values for Lawshe’s content validity ratio: revisiting the original methods of calculation. Measure Evaluat Counseling Dev. 2014;47(1):79–86.

[34] Hinkin TR. A review of scale development practices in the study of organizations. J manag. 1995;21(5):967–88.

[35] Nahm AY, et al. The Q-sort method: assessing reliability and construct validity of questionnaire items at a pre-testing stage. J Modern Appl Statist Methods. 2002;1(1):15.

[36] Royse DD et al., Program evaluation: An introduction. 2010

[37] Polit DF. The content validity index: are you sure you know what’s being reported? Critique and recommendations. Res Nurs Health. 2006;29(5):489–97. 16977646 10.1002/nur.20147

[38] Epstein J, Santo RM, Guillemin F. A review of guidelines for cross-cultural adaptation of questionnaires could not bring out a consensus. J Clin Epidemiol. 2015;68(4):435–41. 25698408 10.1016/j.jclinepi.2014.11.021

[39] Rothbauer P, Triangulation. 2008; 1: 892–894

[40] Nowell LS, et al. Thematic analysis: striving to meet the trustworthiness criteria. Int J Q Methods. 2017;16(1):1609406917733847.

[41] Mills J, Bonner A, Francis KJ. The development of constructivist grounded theory. Int J Q Methods. 2006;5(1):25–35.

[42] Birt L, et al. Member checking: a tool to enhance trustworthiness or merely a nod to validation? Qual Health Res. 2016;26(13):1802–11. 27340178 10.1177/1049732316654870

[43] Onwuegbuzie AJ, Combs JP. Data analysis in mixed research: a primer. Int J Educat. 2011;3:13.

[44] Roth PL. Missing data: a conceptual review for applied psychologists. Personnel Psychol. 1994;47(3):537–60.

[45] Haynes SN, Richard D, Kubany ES. Content validity in psychological assessment: A functional approach to concepts and methods. Psychol Assessm. 1995;7(3):238.

[46] Lynn MR. Determination and quantification of content validity. Nurs Res. 1986;35(6):382–6. 3640358

[47] Philip PM, Kannan SJ. Tool development and validation of the oral cancer patient and diagnostic interval measure. Int J Community Med Public Health. 2022;9(2):819.

[48] Kumar A. Review of the steps for development of quantitative research tools. Adv Pract Nurse. 2015;1(103):10–4172.

[49] Coyle D, Haines A, Lee K. The development of a model validation tool to assist in the conduct of economic evaluations. Can J Health Technol. 2024;4(3):862.

[50] Dźwigoł H. Verification of the need to develop a tool for selecting research methods and techniques. Zeszyty Naukowe Organizacja i Zarządzanie/Politechnika Śląska. 2020;146:87–98.

[51] Burton LJ, Mazerolle SM. Survey instrument validity part I: principles of survey instrument development and validation in athletic training education research. Athletic Train Educat J. 2011;6(1):27–35.

[52] Yaghmaie FJ Content validity and its estimation. 2003; 3(1): 27–35

[53] Almanasreh E, et al. Evaluation of methods used for estimating content validity. Res Soc Administ Pharmacy. 2019;15(2):214–21. 10.1016/j.sapharm.2018.03.066 29606610

[54] Abdollahpour E, et al. The process of content validity in instrument development. Iran Epidemiol. 2010;6(4):66–74.

[55] Rubio DM, et al. Objectifying content validity: conducting a content validity study in social work research. Soc Work Res. 2003;27(2):94–104.

[56] Costello AB, Osborne J. Best practices in exploratory factor analysis: Four recommendations for getting the most from your analysis. Pract Assess Res Evaluat. 2005;10(1):7.

[57] Kaiser HF. An index of factorial simplicity. Psychometrika. 1974;39(1):31–6.

[58] Williams B, Onsman A, Brown T. Exploratory factor analysis: a five-step guide for novices. Austral J Paramed. 2010;8:1–13.

[59] Fabrigar LR, Wegener DT. Exploratory factor analysis. Oxford: Oxford University Press; 2011.

[60] Taherdoost H, et al. Exploratory factor analysis; concepts and theory. Adv Appl Pure Math. 2014;27:375–82.

[61] Maruta T, et al. Regional approach to strengthening biosafety and biosecurity systems in Africa. Global Secur: HealthSci Policy. 2023;8(1):2257766.

[62] Bolarinwa OA. Principles and methods of validity and reliability testing of questionnaires used in social and health science researches. Nigerian Postgraduate Med J. 2015;22(4):195–201. 10.4103/1117-1936.173959 26776330

[63] Alemu T, et al. Evaluation of public health surveillance system performance in Dangila district, Northwest Ethiopia: a concurrent embedded mixed quantitative/qualitative facility-based cross-sectional study. BMC Public Health. 2019;19:1–9. 31640662 10.1186/s12889-019-7724-y PMC6805593

[64] Tsang S, Royse CF, Terkawi AS. Guidelines for developing, translating, and validating a questionnaire in perioperative and pain medicine. Saudi J Anaesthesia. 2017;11(Suppl 1):S80–9. 10.4103/sja.SJA_203_17 PMC5463570 28616007

[65] Frumkin H, et al. Climate change: the public health response. Am J Public Health. 2008;98(3):435–45. 18235058 10.2105/AJPH.2007.119362 PMC2253589

[66] Kruk ME, et al. What is a resilient health system? Lessons from Ebola. The Lancet. 2015;385(9980):1910–2. 10.1016/S0140-6736(15)60755-3 25987159

[67] Whitworth JA, et al. Strengthening capacity for health research in Africa. The Lancet. 2008;372(9649):1590–3. 10.1016/S0140-6736(08)61660-8 PMC2607030 18984193

[68] Sousa VD, Rojjanasrirat W. Translation, adaptation and validation of instruments or scales for use in cross-cultural health care research: a clear and user-friendly guideline. J Evaluat Clin Pract. 2011;17(2):268–74. 10.1111/j.1365-2753.2010.01434.x 20874835

[69] Polit DF, et al. Is the CVI an acceptable indicator of content validity? Appraisal and recommendations. Res Nurs Health. 2007;30(4):459–67. 17654487 10.1002/nur.20199

[70] Grant JS, Davis LL. Selection and use of content experts for instrument development. Res Nurs Health. 1997;20(3):269–74. 9179180 10.1002/(sici)1098-240x(199706)20:3<269::aid-nur9>3.0.co;2-g

[71] Lenz ER. Measurement in nursing and health research. New York: Springer Publishing Company; 2010.

[72] Davis LL. Instrument review: getting the most from a panel of experts. Appl Nurs Res. 1992;5(4):194–7.

[73] Streiner DL. Starting at the beginning: an introduction to coefficient alpha and internal consistency. J Personal Assess. 2003;80(1):99–103. 10.1207/S15327752JPA8001_18 12584072

