# Supplementary material for: Development and validation of an assessment tool for public health emergency management program
Source: Glob Health Res Policy. 2025 Sep 23;10:44. doi: 10.1186/s41256-025-00423-z (PMC12455816; doi:10.1186/s41256-025-00423-z)
Supplement: Supplementary file 1 — Additional file 1. [file 41256_2025_423_MOESM1_ESM.docx]

**Additional file 1: Assessment tool for assessing PHEM program implementation status (60 item).**

| **Part I: Demographic characteristics** | | |
| --- | --- | --- |
| No | **Variable** | **Category** |
| 1 | Sex | 1. Male 2. Female |
| 2 | Age | **………….………….………….** |
| 3 | Level of Education | 1. Degree 2. Masters 3. PhD and above |
| 4 | Years of experience | **…………………….………….** |
| 5 | Region |  |
| 6 | Zone | **………………………….** |
| 7 | Facility type | 1. Health facility 2. District PHEM office 3. Zonal PHEM office 4. Regional PHEM office 5. University 6. National PHEM 7. MOH 8. Other sectors |
| 8 | Staff position | **………………………….** |
| **Part II: PHEM Program Implementation Status Assessment**  **Answer options**: Not at all, To a small extent, Moderately, To a large extent or Fully | | |
| 1. The extent to which established coordination mechanisms have been implemented for collaboration between all sectors/agencies/partners involved in public health emergency management. | | |
| 1. The extent to which full funding required for public health emergency management activities has been secured and disbursed as planned. | | |
| 1. The extent to which the responsible organization has a dedicated, well-equipped, trained team immediately available for conducting prompt outbreak investigations when needed. | | |
| 1. The extent to which protocols are in place to routinely identify communities or areas at high risk of health impacts, based on the specific public health hazard being monitored. | | |
| 1. The extent to which processes exist to systematically recognize population groups particularly vulnerable to health consequences of each public health hazard assessed. | | |
| 1. The extent to which a national policy on public health emergency management has been formally adopted along with a strategic preparedness plan to guide coordinated response activities. | | |
| 1. The extent to which an annual rehearsal/exercise of the public health emergency response plan is regularly scheduled to test coordination and response capabilities. | | |
| 1. To what extent are staff provided with ongoing access to role-specific training tailored to their public health emergency management duties and responsibilities? | | |
| 1. To what extent are all organizational levels of the public health emergency management structure fully staffed according to projected response needs? | | |
| 1. To what extent does a reserve stockpile of essential medical and public health supplies exist to support emergency response operations for at least a three-month period? | | |
| 1. To what extent are the early warning and disease surveillance systems operational and functional? | | |
| 1. To what extent are complete surveillance reports consistently submitted on time to the concerned body according to established timelines? | | |
| 1. To what extent are outbreak investigation reports conducted within the organization according to established protocols and guidelines? | | |
| 1. To what extent are robust monitoring and evaluation systems implemented to track and assess the implementation of PHEM activities and initiatives? | | |
| 1. To what extent are well-established community engagement and public communication mechanisms in place to ensure effective information dissemination and engagement during public health emergencies? | | |
| **Part III: Individual Level characteristics**  **Answer options**: Strongly disagree, Disagree, Neutral, Agree and Strongly agree | | |
| **Capacity building** | | |
| 1. I receive adequate training on implementing the PHEM programs. | | |
| 1. I am motivated to carry out PHEM program activities. | | |
| 1. I feel equipped to carry out PHEM activities effectively. | | |
| 1. Adequate resources are available to me for PHEM implementation. | | |
| **Availability of reinforcement** | | |
| 1. Good incentives are available for me to prioritize PHEM program implementation. | | |
| 1. I am recognized for my contributions to the PHEM program. | | |
| **Engagement** | | |
| 1. I actively participate in PHEM program planning (EPRP - Emergency preparedness response plan). | | |
| 1. The PHEM program integrates input from diverse stakeholders to inform locally appropriate response approaches. | | |
| 1. I receive timely information about PHEM activities and any public health threats. | | |
| 1. Community health workers are actively engaged in supporting the PHEM program. | | |
| **Attitude towards PHEM** | | |
| 1. I perceive PHEM activities as aligned with my values and priorities. | | |
| 1. I believe the PHEM program will positively impact the community's health. | | |
| **Work related factors** | | |
| 1. I can appropriately adapt PHEM activities for local communities. | | |
| 1. Competing priorities overwhelm my capacity for PHEM. | | |
| 1. Coordination across levels allows me to fulfill interconnected PHEM roles. | | |
| **Part IV: Organizational Level characteristics** | | |
| **Information sharing** | | |
| 1. Processes of information sharing and coordination between health facilities on PHEM implementation are good. | | |
| 1. A culture of information sharing, transparency and accountability enables PHEM success. | | |
| 1. Health information systems capture high quality PHEM data. | | |
| **Integration** | | |
| 1. PHEM program activities are well integrated into organizational processes and systems. | | |
| 1. PHEM is well integrated into the broader health system. | | |
| **Planning** | | |
| 1. Adequate program planning and review mechanisms support PHEM implementation. | | |
| 1. Emergency preparedness plans incorporate PHEM program components. | | |
| 1. Surge capacity and business continuity plans account for PHEM needs during crises. | | |
| **Delegation** | | |
| 1. PHEM roles and responsibilities are clearly delineated across different leadership levels. | | |
| 1. Roles and responsibilities clearly defined across sectors involved in PHEM. | | |
| **Availability of resource and funding** | | |
| 1. Adequate funding and budgets are allocated to PHEM activities. | | |
| 1. PHEM financing streams support program sustainability and coverage. | | |
| 1. Procurement and supply chain management enable timely access to necessary PHEM resources. | | |
| 1. National workforce strategies address PHEM human resource requirements. | | |
| 1. Personal protective equipment (PPE) readily available for first responders. | | |
| **Leadership** | | |
| 1. Strong leadership support and commitment exist for the PHEM program. | | |
| 1. Government commitment and leadership enable success of the PHEM program. | | |
| **Effective monitoring and evaluation** | | |
| 1. Effective monitoring and evaluation systems track PHEM implementation progress. | | |
| 1. Regular audits and reviews assess PHEM program effectiveness and identify improvements. | | |
| 1. Ongoing evaluation and learning foster PHEM program adaptation and improvement. | | |
| **Coordination and collaboration** | | |
| 1. Partnerships across organizations effectively support coordinated PHEM implementation. | | |
| 1. Established mechanisms facilitate collaborative planning, supervision and problem-solving for PHEM. | | |
| 1. Stakeholders efficiently work together through clear roles without gaps or conflicts at all levels. | | |
| **Infrastructure** | | |
| 1. Physical infrastructure and technology support effective PHEM implementation. | | |
| 1. Physical infrastructure enables effective national coordination. | | |
| **Legal framework** | | |
| 1. Formal policies adequately define PHEM roles and mandates. | | |
| 1. Supportive policy frameworks comprehensively guide implementation. | | |
| 1. Political environments enable cross-sector support for PHEM. | | |
| 1. Laws and regulations authorize uniform application of protocols nationwide. | | |
| 1. Stakeholders understand legal roles and responsibilities under emergency provisions. | | |

**Additional file 2:** **Assessment tool for assessing PHEM program implementation status (52 items).**

| **Part I: Demographic characteristics** | | |
| --- | --- | --- |
| No | **variable** | **Category** |
| 1 | Sex | 1. Male 2. Female |
| 2 | Age | **………….………….………….** |
| 3 | Level of Education | 1. Degree 2. Masters 3. PhD and above |
| 4 | Years of experience | **…………………….………….** |
| 5 | Region |  |
| 6 | Zone | **………………………….** |
| 7 | Facility type | 1. Health facility 2. District PHEM office 3. Zonal PHEM office 4. Regional PHEM office 5. University 6. National PHEM 7. MOH 8. Other sectors |
| 8 | Staff position | **………………………….** |
| **Part II: PHEM Program Implementation Status Assessment** | | |
| 1. The extent to which established coordination mechanisms have been implemented for collaboration between all sectors/agencies/partners involved in public health emergency management.   **Answer options**: Not at all, To a small extent, Moderately, To a large extent or Fully | | |
| 1. The extent to which full funding required for public health emergency management activities has been secured and disbursed as planned. | | |
| 1. The extent to which the responsible organization has a dedicated, well-equipped, trained team immediately available for conducting prompt outbreak investigations when needed. | | |
| 1. The extent to which protocols are in place to routinely identify communities or areas at high risk of health impacts, based on the specific public health hazard being monitored. | | |
| 1. The extent to which processes exist to systematically recognize population groups particularly vulnerable to health consequences of each public health hazard assessed. | | |
| 1. The extent to which a national policy on public health emergency management has been formally adopted along with a strategic preparedness plan to guide coordinated response activities. | | |
| 1. The extent to which an annual rehearsal/exercise of the public health emergency response plan is regularly scheduled to test coordination and response capabilities. | | |
| 1. To what extent are staff provided with ongoing access to role-specific training tailored to their public health emergency management duties and responsibilities? | | |
| 1. To what extent are all organizational levels of the public health emergency management structure fully staffed according to projected response needs? | | |
| 1. To what extent does a reserve stockpile of essential medical and public health supplies exist to support emergency response operations for at least a three-month period? | | |
| 1. To what extent are the early warning and disease surveillance systems operational and functional? | | |
| 1. To what extent are complete surveillance reports consistently submitted on time to the concerned body according to established timelines? | | |
| 1. To what extent are outbreak investigation reports conducted within the organization according to established protocols and guidelines? | | |
| 1. To what extent are robust monitoring and evaluation systems implemented to track and assess the implementation of PHEM activities and initiatives? | | |
| **Part III: Individual Level characteristics** | | |
| **Capacity building**  **Answer option:** Strongly disagree, Disagree, Neutral, Agree, strongly agree | | |
| 1. I receive adequate training on implementing the PHEM programs. | | |
| 1. I am motivated to carry out PHEM program activities. | | |
| 17. I feel equipped to carry out PHEM activities effectively. | | |
| **Availability of reinforcement** | | |
| 18. Good incentives are available for me to prioritize PHEM program implementation. | | |
| 19. I am recognized for my contributions to the PHEM program. | | |
| **Engagement** | | |
| 20. I actively participate in PHEM program planning (EPRP - Emergency preparedness response plan). | | |
| 1. The PHEM program integrates input from diverse stakeholders to inform locally appropriate response approaches. | | |
| 1. I receive timely information about PHEM activities and any public health threats. | | |
| 1. Community health workers are actively engaged in supporting the PHEM program. | | |
| **Attitude towards PHEM** | | |
| 1. I perceive PHEM activities as aligned with my values and priorities. | | |
| 1. I believe the PHEM program will positively impact the community's health. | | |
| **Work related factors** | | |
| 1. I can appropriately adapt PHEM activities for local communities. | | |
| 1. Coordination across levels allows me to fulfill interconnected PHEM roles. | | |
| **Part IV: Organizational Level characteristics** | | |
| **Information sharing** | | |
| 1. Processes of information sharing and coordination between health facilities on PHEM implementation are good. | | |
| 1. A culture of information sharing, transparency and accountability enables PHEM success. | | |
| **Integration** | | |
| 1. PHEM program activities are well integrated into organizational processes and systems. | | |
| 1. PHEM is well integrated into the broader health system. | | |
| **Planning** | | |
| 1. Emergency preparedness plans incorporate PHEM program components. | | |
| 1. Surge capacity and business continuity plans account for PHEM needs during crises. | | |
| **Delegation** | | |
| 1. PHEM roles and responsibilities are clearly delineated across different leadership levels. | | |
| 1. Roles and responsibilities clearly defined across sectors involved in PHEM. | | |
| **Availability of resource and funding** | | |
| 1. Adequate funding and budgets are allocated to PHEM activities. | | |
| 1. Procurement and supply chain management enable timely access to necessary PHEM resources. | | |
| 1. National workforce strategies address PHEM human resource requirements. | | |
| 1. Personal protective equipment (PPE) readily available for first responders. | | |
| **Leadership** | | |
| 1. Strong leadership support and commitment exist for the PHEM program. | | |
| 1. Government commitment and leadership enable success of the PHEM program. | | |
| **Effective monitoring and evaluation** | | |
| 1. Effective monitoring and evaluation systems track PHEM implementation progress. | | |
| 1. Regular audits and reviews assess PHEM program effectiveness and identify improvements. | | |
| 1. Ongoing evaluation and learning foster PHEM program adaptation and improvement. | | |
| **Coordination and collaboration** | | |
| 1. Partnerships across organizations effectively support coordinated PHEM implementation. | | |
| 1. Stakeholders efficiently work together through clear roles without gaps or conflicts at all levels. | | |
| **Infrastructure** | | |
| 1. Physical infrastructure and technology support effective PHEM implementation. | | |
| 1. Physical infrastructure enables effective national coordination. | | |
| **Legal framework** | | |
| 1. Formal policies adequately define PHEM roles and mandates. | | |
| 1. Political environments enable cross-sector support for PHEM. | | |
| 1. Laws and regulations authorize uniform application of protocols nationwide. | | |
| 1. Stakeholders understand legal roles and responsibilities under emergency provisions. | | |

Additional file 3: Assessment tool for assessing PHEM program implementation status (45 items).

| **Part I: Demographic characteristics** | | |
| --- | --- | --- |
| No | **variable** | **Category** |
| 1 | Sex | 1. Male 2. Female |
| 2 | Age | **………….………….………….** |
| 3 | Level of Education | 1. Degree 2. Masters 3. PhD and above |
| 4 | Years of experience | **…………………….………….** |
| 5 | Region |  |
| 6 | Zone | **………………………….** |
| 7 | Facility type | 1. Health facility 2. District PHEM office 3. Zonal PHEM office 4. Regional PHEM office 5. University 6. National PHEM 7. MOH 8. Other sectors |
| 8 | Staff position | **………………………….** |
| **Part II: PHEM Program Implementation Status Assessment**  **Answer options:** Not at all, To a small extent, Moderately, To a large extent, full | | |
| 1. The extent to which the responsible organization has a dedicated, well-equipped, trained team immediately available for conducting prompt outbreak investigations when needed. | | |
| 1. The extent to which protocols are in place to routinely identify communities or areas at high risk of health impacts, based on the specific public health hazard being monitored. | | |
| 1. The extent to which processes exist to systematically recognize population groups particularly vulnerable to health consequences of each public health hazard assessed. | | |
| 1. The extent to which a national policy on public health emergency management has been formally adopted along with a strategic preparedness plan to guide coordinated response activities. | | |
| 1. The extent to which an annual rehearsal/exercise of the public health emergency response plan is regularly scheduled to test coordination and response capabilities. | | |
| 1. To what extent are staff provided with ongoing access to role-specific training tailored to their public health emergency management duties and responsibilities? | | |
| 1. To what extent are all organizational levels of the public health emergency management structure fully staffed according to projected response needs? | | |
| 1. To what extent does a reserve stockpile of essential medical and public health supplies exist to support emergency response operations for at least a three-month period? | | |
| 1. To what extent are the early warning and disease surveillance systems operational and functional? | | |
| 1. To what extent are complete surveillance reports consistently submitted on time to the concerned body according to established timelines? | | |
| 1. To what extent are outbreak investigation reports conducted within the organization according to established protocols and guidelines? | | |
| 1. To what extent are robust monitoring and evaluation systems implemented to track and assess the implementation of PHEM activities and initiatives? | | |
| **Part III: Individual Level characteristics** | | |
| **Capacity building**  **Answer options**: Strongly disagree, Disagree, Neutral, Agree, strongly agree | | |
| 1. I receive adequate training on implementing the PHEM programs. | | |
| 1. I am motivated to carry out PHEM program activities. | | |
| 1. I feel equipped to carry out PHEM activities effectively. | | |
| **Availability of reinforcement** | | |
| 1. Good incentives are available for me to prioritize PHEM program implementation. | | |
| 1. I am recognized for my contributions to the PHEM program. | | |
| **Engagement** | | |
| 1. I actively participate in PHEM program planning (EPRP - Emergency preparedness response plan). | | |
| 1. The PHEM program integrates input from diverse stakeholders to inform locally appropriate response approaches. | | |
| 1. I receive timely information about PHEM activities and any public health threats. | | |
| 1. Community health workers are actively engaged in supporting the PHEM program. | | |
| **Attitude towards PHEM** | | |
| 1. I perceive PHEM activities as aligned with my values and priorities. | | |
| 1. I believe the PHEM program will positively impact the community's health. | | |
| **Part IV: Organizational Level characteristics** | | |
| **Information sharing** | | |
| 1. Processes of information sharing and coordination between health facilities on PHEM implementation are good. | | |
| **Integration** | | |
| 1. PHEM program activities are well integrated into organizational processes and systems. | | |
| 1. PHEM is well integrated into the broader health system. | | |
| **Planning** | | |
| 1. Emergency preparedness plans incorporate PHEM program components. | | |
| 1. Surge capacity and business continuity plans account for PHEM needs during crises. | | |
| **Delegation** | | |
| 1. PHEM roles and responsibilities are clearly delineated across different leadership levels. | | |
| 1. Roles and responsibilities clearly defined across sectors involved in PHEM. | | |
| **Availability of resource and funding** | | |
| 1. Adequate funding and budgets are allocated to PHEM activities. | | |
| 1. Procurement and supply chain management enable timely access to necessary PHEM resources. | | |
| **Leadership** | | |
| 1. Strong leadership support and commitment exist for the PHEM program. | | |
| 1. Government commitment and leadership enable success of the PHEM program. | | |
| **Effective monitoring and evaluation** | | |
| 1. Effective monitoring and evaluation systems track PHEM implementation progress. | | |
| 1. Regular audits and reviews assess PHEM program effectiveness and identify improvements. | | |
| 1. Ongoing evaluation and learning foster PHEM program adaptation and improvement. | | |
| **Coordination and collaboration** | | |
| 1. Partnerships across organizations effectively support coordinated PHEM implementation. | | |
| 1. Stakeholders efficiently work together through clear roles without gaps or conflicts at all levels. | | |
| **Infrastructure** | | |
| 1. Physical infrastructure and technology support effective PHEM implementation. | | |
| 1. Physical infrastructure enables effective national coordination. | | |
| **Legal framework** | | |
| 1. Formal policies adequately define PHEM roles and mandates. | | |
| 1. Political environments enable cross-sector support for PHEM. | | |
| 1. Laws and regulations authorize uniform application of protocols nationwide. | | |
| 1. Stakeholders understand legal roles and responsibilities under emergency provisions. | | |
